# Blockade of CTLA-4 and Tim-3 pathways induces fetal loss with altered cytokine profiles by decidual CD4^+^T cells

**DOI:** 10.1038/s41419-018-1251-0

**Published:** 2019-01-08

**Authors:** Songcun Wang, Chunqin Chen, Mengdie Li, Jinfeng Qian, Fengyun Sun, Yunyun Li, Min Yu, Mingyan Wang, Xingxing Zang, Rui Zhu, Dajin Li, Meirong Du

**Affiliations:** 1 Laboratory for Reproductive Immunology, NHC Key Lab of Reproduction Regulation(Shanghai Institute of Planned Parenthood Research), Shanghai Key Laboratory of Female Reproductive Endocrine Related Diseases, Hospital of Obstetrics and Gynecology, Fudan University Shanghai Medical College, Shanghai, P.R. China; 2 Reproductive Medicine Center, Hospital of Obstetrics and Gynecology, FudanUniversity Shanghai Medical School, Shanghai, P.R. China; 3 Department of Clinical Laboratory, Hospital of Obstetrics and Gynecology, Fudan University, Shanghai, P.R. China; 4 Department of Medicine, Montefiore Medical Center, Albert Einstein College of Medicine, Bronx, NY USA; 5 The Affiliated Suzhou Hospital of Nanjing Medical University/Suzhou Municipal Hospital, Suzhou, 215008 China

## Abstract

The single and/or combination use of immune checkpoint blockade therapies in human infectious diseases and cancer are rapidly expanding. Despite early efforts, substantial uncertainty remains about the safety and efficacy of immune checkpoint blockade in some populations. Cytotoxic T-lymphocyte-associated protein 4 (CTLA-4) and T-cell immunoglobulin mucin-3 (Tim-3) are the major targetable co-inhibitory receptors on T cells. Here we showed that in animal studies, treatment with either CTLA-4- or Tim-3-blocking antibody caused greater susceptibility to fetal loss with altered cytokine profiles by decidual CD4^+^T (dCD4^+^T) cells. CTLA-4 and Tim-3 pathways appeared to play key roles in maintaining maternal-fetal tolerance by regulating the function of dCD4^+^T cells. In addition, the abnormality in number and functionality of dCTLA-4^+^Tim-3^+^CD4^+^T cells was associated with miscarriage. These findings underscored the important roles of the CTLA-4 and Tim-3 pathways in regulating dCD4^+^T cells function and maintaining normal pregnancy. Our study also emphasized the importance of careful consideration of reproductive safety when choosing immune checkpoint blockade therapies in real world clinical care.

## Introduction

T cell activation following antigen recognition requires a secondary co-stimulatory signal, which can be either positive or negative. Treatment with neutralizing antibodies that target inhibitory signals, or “checkpoint blockade” to enhance immune responses, has been proven as a promising therapeutic strategy for a variety of cancers and chronic viral infections^1^. Cytotoxic T-lymphocyte-associated protein 4 (CTLA-4), programmed death 1 (PD-1), and T-cell immunoglobulin mucin-3 (Tim-3) are the major targetable co-inhibitory receptors on T cells. The development of these immunotherapy agents has increased since the first approval of anti-CTLA-4 therapy (ipilimumab) by the United States Food and Drug Administration for melanoma in 2011^2^. Despite their success, the single use of currently approved antibodies was effective in only 20–30% of patients^3^. Currently, combination approaches against different targets seem to be effective for favorable clinical outcomes^4^. For example, CTLA-4 had a role in both early and late stages of T cell activation and was mainly expressed on T cells residing in lymph nodes^5^, while Tim-3 could exert its function by regulating cell apoptosis^6^, so the combination of anti-CTLA-4 and anti-Tim-3 could restore the greatest degree of T cell function.

During normal pregnancy, the semi-allogeneic fetus has the capacity to avoid immune attack by the maternal immune system, and the placenta is regarded as a pseudo-malignant type of tissue^7^. Impaired tolerance induction or excessive inflammation can lead to severe pregnancy complications such as recurrent spontaneous abortion (RSA), pre-eclampsia, or preterm delivery^8^. T cells, particularly CD4^+^T cells, seem to play a pivotal role in inducing and maintaining maternal-fetal tolerance. Driven by a set of transcriptional regulators and cytokines, naive CD4^+^T helper (Th) cells are able to differentiate into distinct subsets, including Th1, Th2, Th17, and Treg cells^9^. Treg expansion and a polarization toward Th2 bias in the maternal immune response have long been considered the main mechanisms of inducing tolerance toward the fetus^8^. Women who experienced RSA exhibited a marked Th1 bias^10^. The expression of the Th1-type cytokine TNF-α was observed in decidual tissues from failing human pregnancies, and this cytokine was shown to lead to the fetal loss in mice^8^. A lower IL-10 to IFN-γ ratio was associated with abnormal pregnancy outcome in mice, and pregnancy outcomes were improved when Treg cells were transferred from the maternal-fetal interface^11^. Given the similarities between a tumor and a fetus, the effects of checkpoint blockade on the reproductive system and the role of co-signaling molecules in maternal-fetal immunity need to be explored.

A second anti-CTLA-4 monoclonal antibody (mAb), tremelimumab, displayed activity in early phase studies^12^. One anti-Tim-3 mAb (MBG453) was also being investigated in phase I-II clinical trial in patients with advanced malignancies; however, no clinical results have yet been reported^13^. In the present study, efficacy studies of anti-CTLA-4 and anti-Tim-3 were first done in mouse pregnancy models, and then the expression and function of CTLA-4/Tim-3 on CD4^+^T cells during normal pregnancy and miscarriage were explored. The current data demonstrates that combined blockade of the CTLA-4 and Tim-3 pathways results in an increased fetal loss in an experimental mouse pregnancy model by altering the function of decidual CD4^+^T (dCD4^+^T) cells. Furthermore, the co-expression of CTLA-4 and Tim-3 on dCD4^+^T cells is important in Th2 bias and Treg expansion at the maternal-fetal interface, thereby, maintaining a normal pregnancy.

## Results

### Effects of dual blockade of CTLA-4 and Tim-3 on mouse pregnancy

**Fig. 1:**
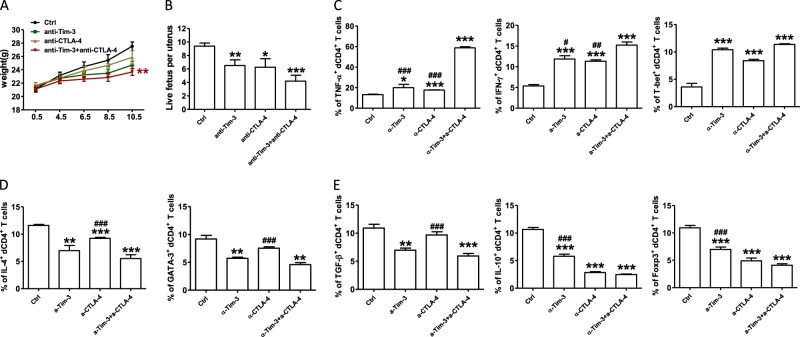
Effects of anti-CTLA-4 or/and Tim-3 antibody during early pregnancy. **a** The weight of pregnant CBA/J females treated with isotype IgG, anti-CTLA-4, anti-Tim-3 antibody, both antibodies i.p. at doses of 500, 250, and 250 mg at days 4.5, 6.5, and 8.5, respectively. **b** The number of live fetuses per uterus from pregnant CBA/J females following treatment with the indicated blocking antibodies. **c**–**e** Quantification of flow cytometric analysis of cytokines production and transcription factors expression by dCD4^+^T cells from pregnant CBA/J female mice following treatment with the indicated blocking antibodies. Data represented the mean ± standard error of the mean (SEM) of *n* = 6–10mice per group and were representative of four independent analyses. *P < 0.05, **P < 0.01, ***P < 0.001, compared with the control group. #*P* < 0.05, ##*P* < 0.01, ###*P* < 0.001, compared with the group of anti-CTLA-4 and anti-Tim-3

In the first assay, we examined pregnant CBA/J females challenged with CTLA4- and/or Tim-3-blocking antibody. Treatment with either blocking antibody caused a higher rate of embryo resorption (data not shown), decreased growth in body weight (Fig. 1a), and reduction in the number of live fetuses per uterus (Fig. 1b). Furthermore, dual blockade of the CTLA4- and Tim-3 pathways had a combined effect, leading to the greatest susceptibility to fetal loss (Fig. 1a, b). These data indicated that CTLA4- and Tim-3-blocking antibody had some side effects on the fertility of mice.

For the checkpoint blockade therapies involved in the regulation of T cell responses, we analyzed the function of dCD4^+^T cells of treated mice to test whether the fetal loss following in vivo blockade of CTLA-4 and/or Tim-3 resulted from CD4^+^T cell dysfunction. The production of Th1-type cytokines, TNF-α, and IFN-γ, and Th1 specific transcription factor, T-bet, increased following single or combined antibody blockade (Fig.1c). While the production of Th2-type and Treg-type cytokines and transcription factors decreased (Fig. 1d, e), treatment with anti-CTLA-4 and/or anti-Tim-3 antibody did not affect the expression of IL-17A and ROR-γt during pregnancy (Figure S1).

### Blockade of CTLA-4 and Tim-3 affected the function of human dCD4^+^T cells

**Fig. 2:**
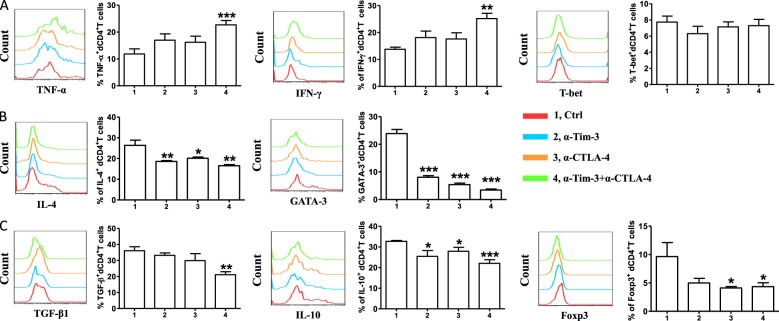
Effect of blocking CTLA-4 and Tim-3 signaling pathways on cytokines production by human dCD4^+^T cells. **a** Expression of Th1-type cytokines and transcription factors of dCD4^+^T cells cultured for 48 h in the presence or absence of anti-CTLA-4 antibody (10 μg/ml), anti-Tim-3 antibody (10 μg/ml), or both. **b** Quantification of flow cytometric analysis of IL-4 and GATA-3 expression by dCD4^+^T cells following treatment with the indicated blocking antibodies. **c** Expression of Treg-type cytokines by dCD4^+^T cells following treatment with the indicated blocking antibodies. Data represented the mean ± SEM. *n* = 12. *P < 0.05, **P < 0.01, ***P < 0.001, compared with the control group

It was impossible to apply anti-CTLA-4 and/or anti-Tim-3 antibody in pregnant women because of ethical constraints. So, in vitro experiments were done using decidual cells obtained from the decidual tissue of normal pregnancies terminated for non-medical reasons. We stimulated dCD4^+^T cells with anti-CD3/CD28 in the presence or absence of antibodies blocking CTLA-4/Tim-3 pathways or combinations of blocking antibodies. After 48 h, expression levels of intracellular cytokines and transcription factors in dCD4^+^T cells were analyzed. Unlike the results observed in infection and cancer^1^, combinations of blocking CTLA-4 and Tim-3 increased the production of Th1-type TNF-α and IFN-γ, but had no effects on the expression of Th1 master transcription factor T-bet (Fig. 2a). Compared with the control group, anti-CTLA-4 or anti-Tim-3 antibody alone was enough to affect the production of Th2-type IL-4 and GATA-3, and this decrease was especially notable in the dual blockade of CTLA-4 and Tim-3 signals (Fig. 2b). As shown in Fig. 2c, the expression of TGF-β1, IL-10 and Foxp3 by dCD4^+^T cells was also down-regulated following combined antibody blockade. However, CTLA-4 or Tim-3 blockade again had no effects on IL-17A production and ROR-γt expression by CD4^+^ T cells at the maternal-fetal interface (Figure S2). Taken together with our in vivo data, dual blocking of CTLA-4 and Tim-3 pathways significantly abrogated Th2 and Treg-mediated fetal protection during normal pregnancy.

### Expression of CTLA-4 and Tim-3 on CD4^+^T cells during early pregnancy

**Fig. 3:**
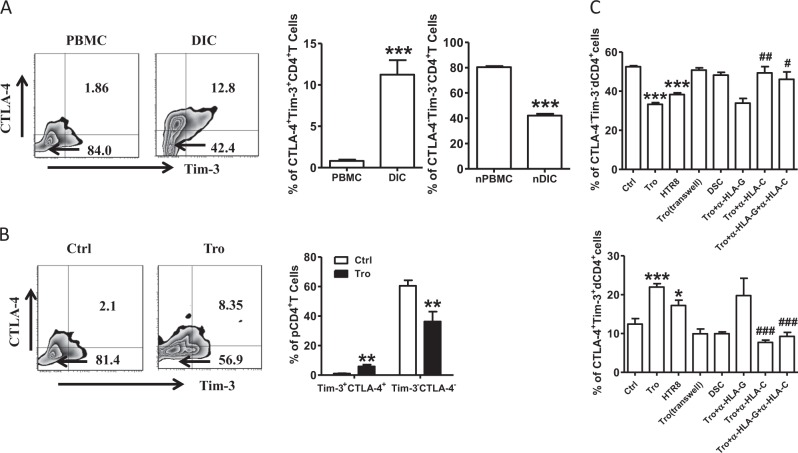
Expression of CTLA-4 and Tim-3 on CD4^+^T cells during human early pregnancy. **a** Flow cytometric analysis (left) and quantification (right) of CTLA-4 and Tim-3 co-expression on gated CD4^+^T cells from peripheral blood mononuclear cells (PBMCs) and decidual immune cells (DICs) during human normal first trimester pregnancies (*n* = 26).***P < 0.001. The flow cytometry plots were representative of three independent experiments. **b** Quantification of flow cytometric analysis of CTLA-4 and Tim-3 co-expression on peripheral CD4^+^T (pCD4^+^T)cells with or without co-culture with trophoblasts (Tros) for 48 h. *n* = 13. ***P < 0.001. **d** Quantification of flow cytometric analysis of CTLA-4 and Tim-3 expression on dCD4^+^T cells cultured alone or co-cultured with equal numbers of Tros (directly or indirectly), or human HTR8/SVneo cells, or decidual stromal cells (DSCs). The α-HLA-G and/or α-HLA-C antibody were used in some wells. *n* = 11, *P < 0.05, **P < 0.01, ***P < 0.001, Compared with the control. ^#^*P* < 0.5, ^##^*P* < 0.01, ^###^*P* < 0.001, compared with the group co-cultured with Tros. Data represented the mean ± SEM

We examined the expression of CTLA-4 and Tim-3 on CD4^+^T cells during the early pregnancy in humans to investigate their potential roles in maternal-fetal immunity. As shown in Fig. 3a, CTLA-4 and Tim-3 were expressed in significantly higher proportions of dCD4^+^T cells than in the peripheral CD4^+^T (pCD4^+^T) cells in human early pregnancy. In contrast, a higher number of CTLA-4^−^Tim-3^−^CD4^+^T cells were observed in the peripheral blood. In addition, co-culture of pCD4^+^T with trophoblasts (Tros) increased the frequency of CTLA-4 and Tim-3 co-expressing cells, while it decreased the frequency of CTLA-4^−^Tim-3^−^CD4^+^T cells (Fig. 3b).

At the maternal-fetal interface, invading placental cells, mainly Tros, maternal-derived decidual stromal cells (DSCs), and decidual immune cells (DICs), come into direct contact with each other^8^. The semi-allogeneic Tros have a unique human leukocyte antigen that expressing a large repertoire of class I HLA-C and nonclassical HLA-G antigens, whereas the class I antigens HLA-A and HLA-B and class II antigens are absent^14,15^. We co-cultured dCD4^+^T cells with different cell populations to further explore the mechanisms mediating the up-regulation of CTLA-4 and Tim-3 expression on dCD4^+^T cells (Fig. 3c). We found that primary Tros and HTR8/Svneo cell line (an immortalized human extravillious trophoblast cell line) but not DSCs could raise the co-expression of CTLA-4 and Tim-3 on dCD4^+^T cells. Separation of Tros and dCD4^+^T cells with a transwell insert canceled the promotion of CTLA-4 and Tim-3 co-expression by Tros. Interestingly, administration of anti-HLA-C antibody, but not anti-HLA-G antibody, significantly inhibited Tros-induced up-regulation of CTLA-4 and Tim-3 co-expression on dCD4^+^ T cells.

### Characterization of dCTLA-4^+^Tim-3^+^CD4^+^T cells during human early pregnancy

**Fig. 4:**
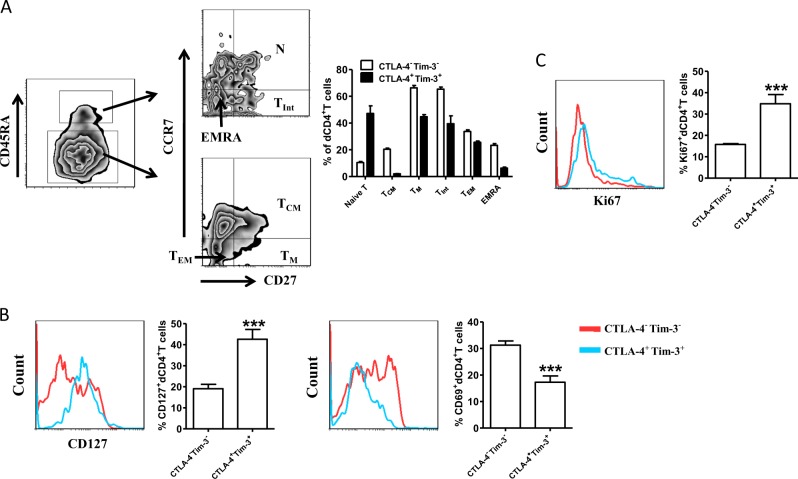
Characterization of Tim-3^+^CTLA-4^+^dCD4^+^T cells during human early pregnancy. **a** The differentiated phenotype of dCTLA-4^+^Tim-3^+^CD4^+^T cells and dCTLA-4^−^Tim-3^−^CD4^+^T cells from human normal early pregnancies (*n* = 12). **b** Expression of CD127 and CD69 on dCTLA-4^+^Tim-3^+^CD4^+^T cells and dCTLA-4^−^Tim-3^−^CD4^+^T cells from the first trimester of normal pregnancies (*n* = 12). **c** Quantification of Ki67 staining in dCTLA-4^+^Tim-3^+^CD4^+^T cells and dCTLA-4^−^Tim-3^−^CD4^+^T cells from human normal early pregnancies (*n* = 12). Data represented the mean ± SEM. ***P< 0.001. A representative dot plot is also shown

Based on CD45RA, CCR7, and CD27 expression, CD4^+^T cells can be classified as naive T cells (CD27^+^CD45RA^+^CCR7^+^), central memory T cells (T_CM_, CD45RA^-^CD27^+^CCR7^+^), memory T cells (T_M_, CD45RA^-^CD27^+^CCR7^−^), T_Int_ (CD45RA^+^CD27^dim^CCR7^−^), effector memory T cells (T_EM_, CD45RA^−^CCR7^−^CD27^−^), and effector memory T cells that express CD45RA cells (EMRA, CD45RA^+^CD27^−^CCR7^−^)^16^. Compared to dCTLA-4−Tim-3^−^CD4^+^T cells, a significant portion of dCTLA-4^+^Tim-3^+^CD4^+^T cells was identified in the naive T cell subset (Fig. 4a), while the concentrations of dCTLA-4^+^Tim-3^+^CD4^+^T cells were lower in central memory and other effector cells. Along with this, the expression of CD127 (as CD4^+^T cells differentiate, they lose expression of CD127) was higher, but the activation marker CD69 was lower in dCTLA-4^+^Tim-3^+^CD4^+^T cells (Fig. 4b). This dCTLA-4^+^Tim-3^+^CD4^+^T cell subset was more likely to survive at the maternal-fetal interface as confirmed by the higher expression of ki67 (Fig. 4c).

**Fig. 5:**
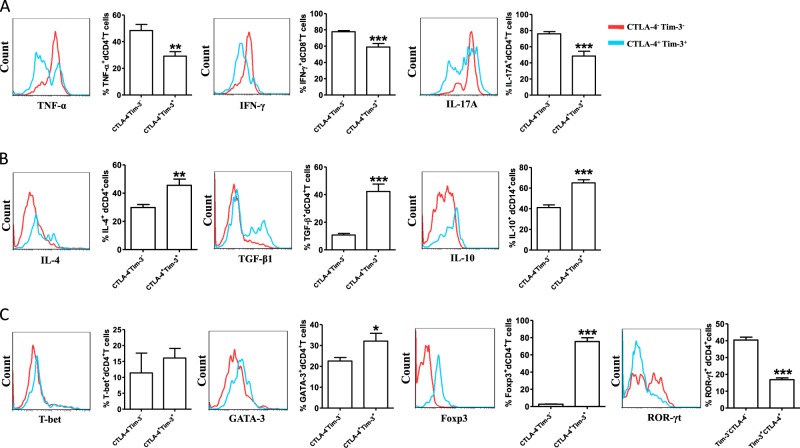
Cytokine production in dCD4^+^T cells during normal pregnancy. **a** Production of the pro-inflammatory cytokines TNF-α, IFN-γ and IL-17A in dCTLA-4^+^Tim-3^+^CD4^+^T cells and dCTLA-4^−^Tim-3^−^CD4^+^T cells from the first trimester of human normal pregnancies. *n* = 9. **b** Quantitation of flow cytometric analysis of IL-4, TGF-β1, and IL-10 of dCTLA-4^+^Tim-3^+^CD4^+^T cells and dCTLA-4^−^Tim-3^−^CD4^+^T cells. *n* = 9. **c** Expression of T-bet, GATA-3, Foxp3, and ROR-γt of dCTLA-4^+^Tim-3^+^CD4^+^T cells and dCTLA-4^-^Tim-3^-^CD4^+^T cells. *n* = 9, Data represented the mean ± SEM. The flow cytometry plots were representative of three independent experiments. *P < 0.05,**P < 0.01, ***P < 0.001, compared with dCTLA-4^-^Tim-3^−^CD4^+^T cells

It has been observed that production of pro-inflammatory or anti-inflammatory cytokines by CD4^+^T cells at the maternal-fetal interface could influence the fate of pregnancy^17^. Next, we evaluated whether dual CTLA-4 and Tim-3 expression correlated with the dCD4^+^T cells function of producing cytokines. We found that the production of pro-inflammatory cytokines (TNF-α, IFN-γ, and IL-17A) by dCTLA-4^+^Tim-3^+^CD4^+^T cells was lower than that by dCTLA-4^−^Tim-3^−^CD4^+^T cells (Fig. 5a). Additionally, dCTLA-4^+^Tim-3^+^CD4^+^T cells are associated with increased Th2-type and Treg-type cytokines production (Fig. 5b). We then examined the expression of master transcription factors associated with Th1/Th2/Treg/Th17 cells. As shown in Fig. 5c, dCTLA-4^+^Tim-3^+^T cells exhibited significantly higher GATA-3 and Foxp3, but lower ROR-γt expression. However, the expression of T-bet did not change with the expression of CTLA-4 and Tim-3 on dCD4^+^T cells. These data gave the first indication that dCD4^+^T cells characterized by differential expression of CTLA-4 and Tim-3 contained cells in different functional states.

### Disorder of the number and function of dCTLA-4^+^Tim-3^+^CD4^+^T cells in miscarriage

**Fig. 6:**
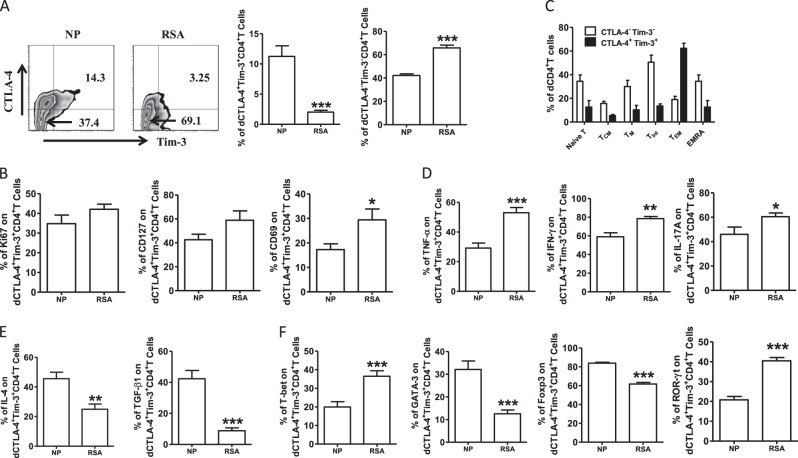
Decreasednumberof dCTLA-4^+^Tim-3^+^CD4^+^Tcells with disordered function in patients with recurrent spontaneous abortion. **a** Frequency of dCTLA-4^+^Tim-3^+^CD4^+^Tcells from normal pregnancy (NP) and patients diagnosed with recurrent spontaneous abortion (RSA). **b** Expression of ki67, CD127, and CD69 of dCTLA-4^+^Tim-3^+^CD4^+^T cells from NP and RSA. **d**–**f** Pro- and anti-inflammatory cytokines production and transcription factors expression by dCTLA-4^+^Tim-3^+^CD4^+^Tcells from NP and RSA. Data represented the mean ± SEM. *P < 0.05, **P < 0.01, ***P < 0.001. NP normal pregnancy, *n* = 23; RSA recurrent spontaneous abortion, *n* = 26

**Fig. 7:**
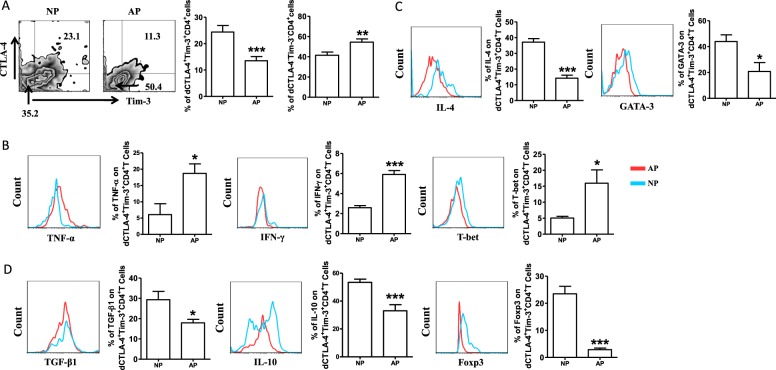
Altered frequency and function of dCTLA-4^+^Tim-3^+^CD4^+^T cells in mouse abortion-prone model. **a** Frequency of dCTLA-4^+^Tim-3^+^CD4^+^T cells from normal pregnancy (NP) and abortion-prone (AP) mice. **b**–**d** Pro- and anti-inflammatory cytokines production and transcription factors expression by dCTLA-4^+^Tim-3^+^CD4^+^T cells from NP and AP mice assessed by flow cytometric analysis. Data represented the mean ± SEM. *P < 0.05, **P < 0.01, ***P < 0.001. NP normal pregnancy, *n* = 12; AP abortion prone, *n* = 14

As blockade of CTLA-4 and Tim-3 signals was harmful to the maintenance of normal pregnancy, and dCTLA-4^+^Tim-3^+^CD4^+^T cells were associated with Th2-type and Treg-type cytokines production, we further explored the possible clinical significance of CTLA-4 and Tim-3 by analyzing samples from normal pregnant subjects and patients who experienced RSA. Flow cytometric analysis revealed a much lower co-expression of CTLA-4 and Tim-3 on dCD4^+^T cells from RSA compared with that from normal pregnancy (Fig. 6a). Although the expression of Ki67 and CD127 on dCTLA-4^+^Tim-3^+^CD4^+^T cells was stable in RSA, these cells expressed significantly higher levels of CD69 (Fig. 6b). A significant portion of dCTLA-4^+^Tim-3^+^CD4^+^T cells was identified in the naive T cell subset (Fig. 4a) in normal pregnancy. In contrast, the proportion of dCTLA-4^+^Tim-3^+^CD4^+^T cells in the T_Naive_ subset was limited from RSA, but was greater in the T_EM_ subset (Fig. 6c). In addition, dCTLA-4^+^Tim-3^+^CD4^+^T cells from RSA expressed higher levels of TNF-α, IFN-γ, IL-17A, T-bet, and ROR-γt (Fig. 6d, f), but lower amounts of IL-4, TGF-β1, GATA-3, and Foxp3 (Fig. 6e, f). We could not detect the difference in IL-10 expression on dCTLA-4^+^Tim-3^+^CD4^+^T between normal pregnancy and RSA (Figure S3). We also observed fewer dCTLA-4^+^Tim-3^+^CD4^+^T cells and higher expression of Th1-type cytokines, but lower Th2-type and Treg-type cytokines by dCTLA-4^+^Tim-3^+^CD4^+^T cells in female CBA/J mated with male DBA/2 mice, a well-established model of abortion (Fig. 7).

## Discussion

There is evidence that T cells lose their effector functions on chronic stimulation by tumors or virus antigens, which is accompanied by aprogressive increase in the inhibitory receptors expressed on them, including CTLA-4 and Tim-3^18^. Given that Tim-3 negatively regulates IFN-γ-mediated Th1 responses, the use of anti-Tim-3 antibody may complement therapies (like anti-CTLA-4 antibody) relieving T cell anergy/exhaustion/tolerance pathways^19^. At the 2010 American Society of Clinical Oncology Annual Meeting, ipilimumab(an anti-human CTLA-4 antibody) was reported to extend the overall survival in patients with advanced melanoma by 10months, and an important study showed considerable therapeutic benefit in combining anti-CTLA-4 and anti-Tim-3 to control CT26 colon adenocarcinoma^20^. Combination therapies were thought to be a promising novel therapeutic approach for several types of human malignancies^13^. Iilimumab was categorized as pregnancy category C by the United States Food and Drug Administration^21^, due to the less clear role of the CTLA-4 axis in fetal immunity. One anti-Tim-3 mAb is being investigated in a phase I-II clinical trial, but no clinical results have yet been reported^13^. The purpose of the present study was to examine whether blocking these inhibitory pathways affected the outcome of pregnancy and determine whether CTLA-4 and Tim-3 pathways were involved in the establishment and maintenance of maternal-fetal tolerance.

Pregnant CBA/J females treated with Tim-3-and/or CTLA-4-blocking antibodies became more susceptible to fetal loss (Fig. 1), although the further development of any surviving embryos needs additional study. With unknown risks of harm to mothers and the potential of birth defects, use of CTLA-4 and/or Tim-3 blockade agents in pregnancy would likely pose great risk of abortion, and it would ultimately be an individualized decision made with careful consideration of potential benefits and risks.

Upon encountering antigens displayed by antigen presenting cells or being driven by a set of cytokines, naive CD4^+^T cells can differentiate into distinct subsets, including Th1, Th2, Th17, and Treg cells. Their differentiation is controlled by the following lineage-specific master transcription factors: T-bet for Th1, GATA-3 for Th2, ROR-γt for Th17, and Foxp3 for Treg^22,23^. The production of pro- or anti-inflammatory cytokines at the maternal-fetal interface influences the outcome of pregnancy because there is dominance by Th2-type and Treg-type cytokines (IL-4, IL-10, TGF-β1) during normal pregnancy and dominance by Th1-type cytokines in miscarriage^8,24^. For example, committed Th1 polarization blocking Treg differentiation triggers antigen specific fetal loss^25^. We found that single or combination of CTLA-4 and Tim-3 blockade resulted in a decreased production of Th2-type and Treg-type cytokines, but it increased Th1-type cytokines of dCD4^+^T cells both in vivo and in vitro. These data indicate that fetal loss induced by CTLA-4 and/or/ Tim-3 blockade maybe associated with the dysfunction of CD4^+^T cells by the production of disordered cytokines.

CD4^+^T cells are thought to play a pivotal role in inducing and maintaining maternal-fetal tolerance, and for controlling maternal viremia^26^. However, the mechanism that regulates CD4^+^T cell response during pregnancy remains to be explored. We found CTLA-4 and Tim-3 were expressed on significantly higher proportions of dCD4^+^T cells than pCD4^+^T cells. In addition, Tros contribute to the expansion of dCTLA-4^+^Tim-3^+^CD4^+^T cells depending on HLA-C in a direct contact manner. Furthermore, dCTLA-4^+^Tim-3^+^CD4^+^T cells possessed higher proliferative capability and produced more Th2-cytokines and Treg cytokines to further promote maternal-fetal tolerance. Tros, not DSCs, up-regulated the percentage of dCTLA-4^+^Tim-3^+^CD4^+^T cells, providing further evidence that embryonic Tros have the unique ability to instruct DICs to develop a regulatory phenotype for fetal tolerance. Our data also confirmed that HLA-C expressed on Tros was involved in the regulation of dCD4^+^T cells function, in addition to eliciting a direct cytotoxic response by CD8^+^ T cells^27,28^. CTLA-4 and Tim-3 are well-known inhibitory co-stimulatory signals that contribute to the exhaustion of T cells^18^. Based on their predicted ability to survive and proliferate, T cell subpopulations can be ranked (from highest to lowest) as follows: Naive → T_CM_ → T_M_ → T_Int_ → T_EM_ → EMRA^29^. Our results indicated that CTLA-4 and Tim-3 co-expression demarcated a T_Naive_ phenotypic signature that favored persistence compared to dCTLA-4^−^Tim-3^−^CD4^+^T cells. Differentiation of T cells into effector cells during primary immune responses has important consequences for the development of active immunity, the proportion of T_EM_ subset was higher in dCTLA-4^+^Tim-3^+^CD4^+^T cells in RSA (Fig. 6c). Along with this, the production of Th1- and Th17-type cytokines by dCTLA-4^+^Tim-3^+^CD4^+^T cells also increased in RSA. There was compelling evidence showing an alteration of Th1/Th2/Th17 and Treg cells subsets in unexplained recurrentmiscarriages^30^. Together with the decreased cell number, proliferation and anti-inflammatory production by dCTLA-4^+^Tim-3^+^CD4^+^T cells in RSA, CTLA-4, and Tim-3 pathways were associated with dCD4^+^T cell function and pregnancy outcome. Though the expression of IL-17A and ROR-γt varied in the dCTLA-4^+^Tim-3^+^CD4^+^T cells and dCTLA-4^−^Tim-3^−^CD4^+^T cells, Th17-type cytokines possessed weak reactivity to CTLA-4 and/or Tim-3 blockade. Thus, Th17 immune responses may not function along with CTLA-4 and Tim-3 during pregnancy.

The single and/or combination use of immune checkpoint blockade therapies in human infectious diseases and cancer is rapidly expanding^1^. The results of the current study show that CTLA-4 and Tim-3 pathways appear to play key roles in maintaining maternal-fetal tolerance by regulating the function of dCD4^+^T cells. The abnormality in number and functionality of dCTLA-4^+^Tim-3^+^CD4^+^T cells is at least one of the reasons leading to the occurrence of miscarriage. In animal studies, anti-CTLA-4/Tim-3 antibody clearly increases the risks of abortion. Thus, characterizing the efficacy and safety of anti-CTLA-4/Tim-3 antibody (and other immune checkpoint inhibitors) in different patient populations is a critical objective. Reproductive safety must be considered, especially when these therapies are used during pregnancy.

## Materials and methods

### Mice

CBA/J female, DBA/2 male, and BALB/c male mice were purchased from Huafukang (Beijing, China). All of the animals were conducted in accordance with the National Guidelines for Animal Care and Use in Research (China). The experimental methods in particular were carried out in accordance with the approved guidelines. Eight-week-old CBA/J females were mated to BALB/c males to induce normal pregnancies, CBA/J females were mated to DBA/2 males to establish abortion-prone models, and inspected every morning for vaginal plugs. The day of visualization of a plug was designated as day 0.5 of pregnancy. In some experiments, pregnant females received injections of single or combined anti-CTLA-4 antibody (clone 9H10, BioLegend, U.S.A.), anti-Tim-3 antibody (clone RMT3-23, BioLegend, U.S.A.), or isotype IgG; i.p. at doses of 500, 250, and 250 mg on days 4.5, 6.5, and 8.5, respectively, based on our previous publications^31,32^. All pregnant mice were monitored at day 10.5 of pregnancy.

### Preparation of mouse cells

Uteri from pregnant mice were dissected free from the mesometrium and the fetal and placental tissues were carefully removed from the uterus. Washed and Minced uteri were digested in RPMI 1640 (HyClone, U.S.A.) supplemented with collagenase type IV (1.0 mg/ml, Worthington Biomedical, U.S.A.) and DNase I (150 U/ml, Applichem, Germany) for 30 min at 37 °C with gentle agitation. Cells were cultured in RPMI 1640 supplemented with 10% FBS, 100 U/ml penicillin, 100 μg/ml streptomycin, and 1 μg/ml amphotericin B at 37 °C in 5% CO_2_ for 2 h to remove adherent stromal cells. Then the cell suspensions were collected and Phorbol 12-myrstate 13-acetate (PMA) (50 ng/ml, BioLegend, U.S.A.), ionomycin (1 μg/ml, Biolegend, U.S.A.) and brefeldin A (10 mg/ml, BioLegend, U.S.A.) were added in the culture for 4 h for intracellular cytokine analysis.

### Human samples

This study was approved by the Human Research Ethics Committee of the Obstetrics and Gynecology Hospital, Fudan University. Every participant signed a written informed consent form. Samples of human first-trimester pregnancies were obtained from clinically normal pregnancies (terminated for non-medical reasons, *N* = 72, whole peripheral blood, villous and decidual tissues) and miscarriages (diagnosed as recurrent spontaneous abortion, RSA, and excluding those resulted from chromosomal defects, endocrine, anatomic, genetic abnormalities, infection, etc., *N* = 26, decidual tissues). Samples were immediately collected for the isolation of peripheral blood mononuclear cells (PBMCs), Tros, DSCs, and DICs.

### Human cell isolation

PBMCs were isolated from peripheral blood samples of normal pregnancies using Ficoll density gradient centrifugation (Huajing, China). Tros were isolated from the normal placenta tissues by trypsin-DNase I (150 U/ml, Applichem, Germany) digestion and discontinuous Percoll gradient centrifugation (GE Healthcare, U.S.A.). DICs and DSCs were obtained from the normal or RSA decidual tissue digesting in RPMI 1640 (HyClone, U.S.A.) supplemented with collagenase type IV (1.0 mg/ml, CLS-1, Worthington Biomedical, U.S.A.) and DNase I (150 U/ml, Applichem, Germany) as described previously^31,33^. CD4^+^T cells were isolated by magnetic affinity cell sorting using CD4 microbeads (MiltenyiBiotec, Germany).

### Co-culture of CD4^+^T cells and other cells

Freshly isolated Tros (Matrigel-coated 24-well plates) or HTR8/SVneo cells, or DSCs were seeded at a density of 2 × 10^5^ cells/ml per well in 24-well plates overnight. The cells were then washed with PBS (HyClone, U.S.A.). Equal numbers of dCD4^+^T cells or pCD4^+^T cells were added to each well. In some wells, dCD4^+^T cells were plated in the upper chamber (0.4 mm poresize cell culture inserts, Millipore, Germany), while Tros were plated in the lower chamber to establish indirect cell contact. In some wells, anti-HLA-G (10 μg/ml, clone 87G; Biolegend, U.S.A.) or/and anti-HLA-C (10 μg/ml, clone W6/32; Biolegend, U.S.A.) were added. PMA (50 ng/ml), ionomycin (1 μg/ml) and brefeldin A (10 mg/ml) were added 4 h before the end of the 48 h culture for intracellular cytokine analysis. The immune cells were then harvested for flow cytometry analysis.

### CTLA-4 and Tim-3 targeting experiments

dCD4^+^T cells were cultured (5 **×** 10^5^ per well) in the presence of anti-CTLA-4 (10 μg/ml, clone L3D10, BioLegend, U.S.A.), anti-Tim-3 (10 μg/ml, clone F38-2E2, BioLegend, U.S.A.), both of the two antibodies, or isotype control for 48 h. Brefeldin A, PMA, and ionomycin was added 4 h before the end of the culture. The cells were then collected for further analysis by flow cytometry.

### Flow cytometry

Cell surface molecular expression and intracellular cytokines production were evaluated using flow cytometry. FITC-conjugated anti-human or anti-mouse CD4, AlexaeFluor® 488- conjugated anti-human or anti-mouse Foxp3, IFN-γ, PE-conjugated anti-human Tim-3, or anti-mouse Tim-3, T-bet or GATA-3, PE/CY7-conjugated anti-human IL-10, TNF-α, IFN-γ, IL-17A, TGF-β1 or CD45RA, PerCP/Cy5.5-conjugated anti-human CCR7, PerCP/Cy5.5-conjugated anti-mouse GATA-3, T-bet, or IL-17A, APC-conjugated anti-human CTLA-4, GATA-3, or anti-mouse CTLA-4, TNF-α, IL-10 or GATA-3, Brilliant Violet 421-conjugated anti-human CD27, Ki-67, ROR-γt, or IL-4, or anti-mouse CD4, Tim-3, IFN-γ, IL-4, or TGF-β1, Brilliant Violet 510-conjugated anti-human CD4, anti-mouse TNF-α, IL-4 or IFN-γ antibodies (Biolegend, U.S.A.) were used. For intracellular staining, cells were fixed and permeabilized using the Fix/Perm kit (Biolegend, U.S.A.). Flow cytometry was performed on a Beckman-Coulter CyAn ADP cytometer and analyzed with FlowJo software (Tree Star, U.S.A.).

### Statistical analysis

The statistical significance of differences between two groups was determined by the post-hoc Dunnett *t*-test. Multiple groups were analyzed with GraphPad Prism Version 7 by one-way or two-way ANOVA with Bonferroni post *t*-tests. For all statistical tests, *p* values < 0.05 were considered statistically significant.

## Supplementary information

Supplementary Figures

Supplementary figure legends
