# Supplementary figures and images for: Blockade of CTLA-4 and Tim-3 pathways induces fetal loss with altered cytokine profiles by decidual CD4+T cells
Source: Cell Death Dis. 2019 Jan 8;10(1):15. doi: 10.1038/s41419-018-1251-0 (PMC6325160; doi:10.1038/s41419-018-1251-0)

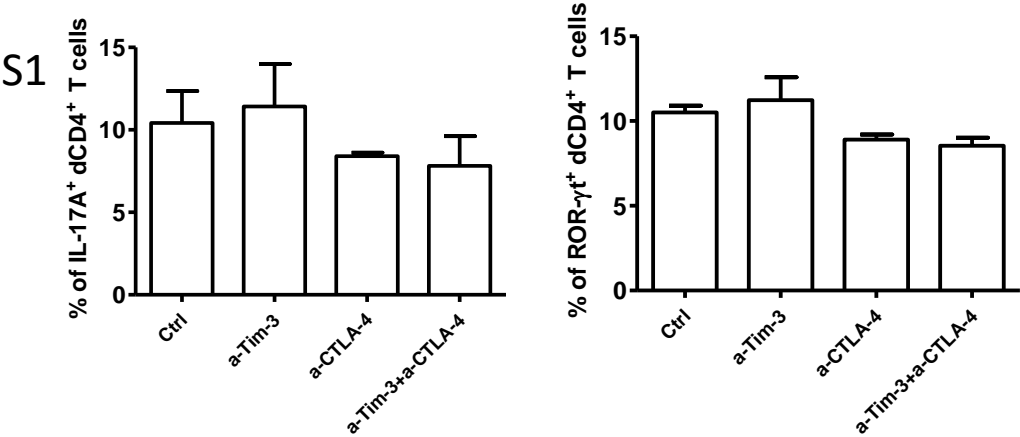

human

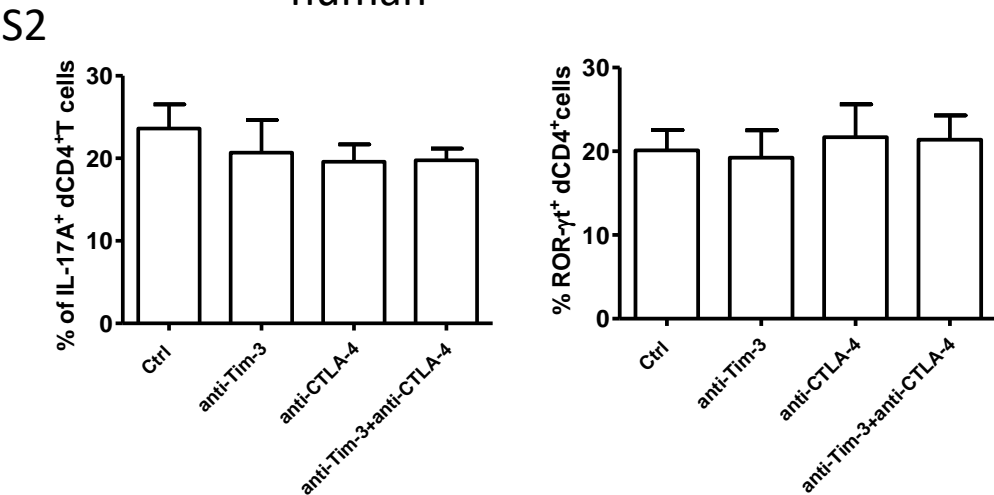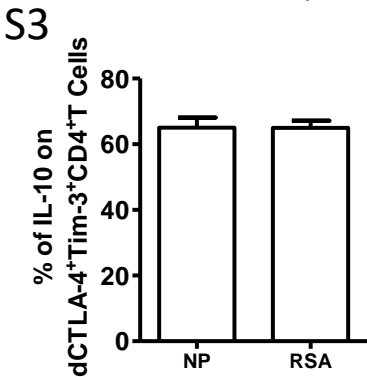

Supplement: Supplementary file 1 — Supplementary Figures [file 41419_2018_1251_MOESM1_ESM.pdf]
