# Supplementary material for: Blockade of CTLA-4 and Tim-3 pathways induces fetal loss with altered cytokine profiles by decidual CD4+T cells
Source: Cell Death Dis. 2019 Jan 8;10(1):15. doi: 10.1038/s41419-018-1251-0 (PMC6325160; doi:10.1038/s41419-018-1251-0)
Supplement: Supplementary file 2 — Supplementary figure legends [file 41419_2018_1251_MOESM2_ESM.docx]

**Figure S1.**Quantification of flow cytometric analysis of IL-17A and ROR-γt expression by dCD4^+^ T cells of pregnant CBA/J female mice treated with isotype IgG, anti-CTLA-4, anti-Tim-3 antibody, both antibodies i.p. at doses of 500, 250, and 250 mg at days 4.5, 6.5, and 8.5, respectively. Data represented the mean ± SEM, n=6-10 mice per group.

**Figure S2.** Expression of IL-17A and ROR-γt of dCD4^+^ T cells cultured for 48h in the presence or absence of anti-CTLA-4 antibody (10 μg/ml), anti-Tim-3 antibody (10 μg/ml), or both. Data represented the mean ± SEM. n=12.

**Figure S3.** IL-10 production and transcription factor expression by dCTLA-4^+^Tim-3^+^CD4^+^ T cells from normal pregnancy (NP) and recurrent spontaneous abortion (RSA). Data represented the mean ± SEM. NP, normal pregnancy, n=23; RSA, recurrent spontaneous abortion, n=26.
